# Delay in reaching health facilities for emergency obstetric care and associated factors among postpartum mothers at Bale zones, Ethiopia. A cross-sectional study

**DOI:** 10.1371/journal.pgph.0002964

**Published:** 2024-02-28

**Authors:** Derese Eshetu, Zeleke Aschalew, Agegnehu Bante, Belda Negesa, Degefa Gomora, Neway Ejigu, Girma Geta, Abera Mersha

**Affiliations:** 1 Department of Midwifery, College of Medicine & Health Sciences, Madda Walabu University, Goba, Ethiopia; 2 School of Nursing, College of Medicine & Health Sciences, Arba Minch University, Arba Minch, Ethiopia; 3 Department of Midwifery, Institute of Health Sciences, Bule Hora University, Bule Hora, Ethiopia; International Centre for Diarrhoeal Disease Research, Bangladesh (icddr,b), BANGLADESH

## Abstract

Maternal mortality occurs in developing nations as a result of inadequate health care, delayed medical attention and the inability to access medical facilities. The three-delay model was employed to determine the causes of maternal death. There was limited data on maternal delay in reaching health facilities for emergency obstetric care services in the study area. Therefore, the aim of this study was to assess the prevalence of delay in reaching health facilities for emergency obstetric care and associated factors among postpartum mothers at Bale and east Bale zones. An institutional-based cross-sectional study was conducted among 407 postnatal women from April 6 to May 6, 2022. A systematic sampling technique was used to select study participants. The data were collected electronically using an Open Data Kit and exported to SPSS window version 25 for cleaning and analysis. Both bivariable and multivariable analysis was done by using a binary logistic regression model to identify factors associated with delay in traveling for emergency obstetric care services. Statistical significance was declared at P-value < 0.05. In this study, the prevalence of delay in reaching health facilities during emergency obstetric care was 38.1% (95%CI: 33.3, 43). The following factors showed significant association with delay in reaching health facilities during emergency obstetric care: Average monthly income (AOR = 1.87; 95% CI: 1.12, 3.14), distance (AOR = 4.35; 95% CI: 2.65, 7.14), a referral from other health facilities (AOR = 1.77; 95% CI: 1.01, 3.11) and delay one (AOR = 2.9; 95% CI: 1.7, 4.93). This study showed that the prevalence of delay in reaching health facilities for emergency obstetric care services in the study area was high. Promoting road accessibility and transport mechanisms and strengthening referral mechanisms shall be emphasized.

## 1. Background

Maternal delays for emergency institutional delivery have a significant impact on maternal mortality. Globally, one every two minutes, women die each day from complications related to pregnancy and childbirth. Of these, sub-Saharan Africa (SSA) alone accounted for 70% of maternal mortality [1]. According to the Ethiopian Demographic Health Survey (EDHS) report, the maternal mortality rate (MMR) in Ethiopia is 412 deaths per 100,000 live births, in which maternal delay is one of the most contributing factors for maternal death [2].

Maternal delays in reaching health facilities are defined as the time interval between making the decision to obtain emergency obstetric care and the initiation of early health care services [3]. The first delay occurs at the family, household and community levels. It indicates a decision to seek treatment for pregnancy-related complications. The second delay reveals a delay in reaching the health facilities that provide emergency obstetric care services, which is the most common factor in the rural areas and the third delay reflects delay in receiving appropriate treatment at the health facility [3, 4].

Delay in reaching health facilities during emergency obstetric care is identified as a contributing factor to maternal morbidity and mortality in Ethiopia. Studies have shown that women suffering from delay during institutional deliveries have encountered a number of health problems, including postpartum hemorrhage, antepartum hemorrhage, uterine rupture, premature rupture of membranes and obstetric fistulas [5–7]. The majority of these complications are preventable; however, delay during obstetric emergency care can seriously harm the outcome of pregnancy [6].

According to the findings from 700 maternal death review reports, delay in reaching health facilities was responsible for 24 percent of maternal mortality. Hence, the main cause of maternal delay two is delayed arrival at a referred facility [8]. The prevalence of delay in reaching health facilities during emergency obstetric care was different in developing countries. A study showed that the prevalence of mothers experiencing delay two was 39.6%, 50.2%, and 65% [9–11]. A study done in Ethiopia revealed that the prevalence of delay in reaching health facilities in utilizing institutional delivery ranged from 29.7% to 59.7% [12–14].

The Ethiopian government has taken a lot of measures to tackle the problem of high maternal delay in reaching health facilities during institutional delivery services through different activities to improve community demand for increased access and service utilization. By availing ambulance service to reach health facilities for childbirth, community mobilization campaigns, user-fee exemption for institutional delivery service, maternal waiting home service, pregnant women conferences and early antenatal care initiation by connecting with the health extension program [2, 3, 8].

According to available evidences, distance, poverty, decision-making power, lack of information, perceived inequality of care at health facilities, poor-quality services, cultural beliefs and practices and geographical inaccessibility were identified as barriers contributing to delay in reaching health facilities during emergency obstetric care [3, 7, 12, 13]. Most of the former studies were done before the occurrence of the coronavirus disease 2019 (COVID-19), which was a great challenge for the health care delivery system. There was insufficient data about the prevalence of maternal delay in reaching health facilities for emergency obstetric care in a study area.

Therefore, the aim of this study was to investigate the prevalence and factors associated with delay in reaching health facilities for emergency obstetric care among postpartum mothers at public hospitals of Bale and East Bale zones, Oromia region, south eastern Ethiopia in 2022.

## 2. Methods and materials

### Study settings, study design and period

The Bale and East Bale zones were found in the Oromia regional state in southeast Ethiopia. The zone had consisted of twenty one districts, of which three were urban administrative centers and of the rural districts, nine were agrarian and nine were agro-pastoralist. The zone had a total population of 1,888,366 of which 936,630 were female. All six public hospitals were found in the zones, namely: Madda Walabu Primary Hospital, Delo Mena General Hospital, Ginir General Hospital, Goro Primary Hospital, Robe General Hospital and Madda Walabu University Goba Referral Hospital (MWUGRH). A cross-sectional study design was conducted from April 6^th^ to May 6^th^, 2022.

### Population

The source population were all women who gave birth in public hospitals of Bale and East Bale zones, whereas the study population were all women who gave birth during the data collection period in public hospitals of Bale and East Bale zones.

### Eligibility criteria

All mothers who gave birth in the Bale and East Bale zones public hospitals were included in the study, while mothers who were severely sick and unable to respond during the data collection period were excluded.

### Sample size and sampling procedure

The sample size was calculated using the single population proportion formula and the StatCal application of Epi-Info version 7.2.5. The required sample size for this study was determined using the following assumptions: desired precision (d)  =  5%, confidence level  =  95% (Zα/2  =  ±1.96 value) and the prevalence of mothers who experienced delay during emergency obstetric care was 59.7% [14]. Thus, the final calculated sample size with a 10% non-response rate was 407.

The six hospitals found in the Bale and East Bale zones were included in the study. The average source population of each hospital was taken by reviewing the previous year’s similar-month delivery report in a data collection period. The proportional allocation were done for six hospitals to collect 407 sample. The study participants was systematically selected with an interval of K every three persons from each hospital. Finally, the total sample size required was collected within the given period.

### Data collection tools and procedures

A structured questionnaire that was administered by an interviewer was adapted from the survey instruments constructed by the Johns Hopkins Program for International Education in Gynecology and Obstetrics (JHPIEGO) maternal and neonatal health program [15] and from an earlier literature review [3, 12–14, 16]. The questions are divided into three sections: transportation related variables, obstetric related factors and socio demographic characteristics. A pre-test was done on twenty one respondents (five percent of the sample size) at Dodola General Hospital (outside the study area) to check skip patterns, phrases, clarity, logical sequence and culturally unacceptable issues of the questions. The questionnaire was collected by ODK (Open Data Kit) collect.

Six nurses with bachelor’s degrees were hired for data collection and three bachelor’s-degree health officers were recruited as supervisors. The purpose of the study was explained and training was given for the data collectors and supervisors for one day on using the ODK application, connecting to the server, saving and sending files. On the data collector’s smart phone, the ODK Collect version 1.17.2 software was installed and the blank form was downloaded from the server. After that, a week prior to the actual data collection, the tool was pre-tested. Supervisors regularly checked the data collection technique by staying in contact with the data collectors. The data was collected through face-to-face interviews with postpartum mothers in private places while discharged.

### Study variables

#### Dependent variable

Delay in reaching health facilities

#### Independent variables

Socio-demographic factors: Maternal age, residence, marital status, mother’s education, husband education, mother’s occupation, husband occupation and house hold monthly income.

Obstetrics related factors: Gravidity, number of children, type of pregnancy (single or multiple), birth readiness, mode of delivery, history of home delivery, place came from to delivery, current pregnancy outcome, birth weight and type of complication.

Transportation related factors: Distance, means of transportation, road availability, public transport available and transportation problem.

### Operational definitions

Delay in reaching health facilities: was time difference from starting to reach health facilities after decision has made. Time taken ≥1 hour to reach facility considered as delay and less than an hour considered no delay [13]

Emergency obstetric care: Immediate medical or surgical care provided to a woman who was in labor or had just given birth [12].

### Data quality control

A structured questionnaire conducted by interviewers was first developed in English, then translated into Afan Oromo and Amharic versions by language experts and retuned to English for consistency. A pretest was conducted to ensure the clarity of the tool. The training was given to data collectors and supervisors on utilizing ODK for data collection, along with a comprehensive explanation of each question present in the tool. Supervisors reviewed and checked the questionnaires to ensure the completeness of the forms. To prevent social desirability bias, each woman was interviewed in a separate private room. Additionally, the investigators were maintained constant contact with the server to verify the files sent from every data collector.

### Data processing and analysis

The data collector’s smart mobile phone sent each data file, which was downloaded and saved as an excel file from the server. The data set was imported to SPSS 25 versions for cleaning, coding and analysis. Descriptive statistics such as mean, standard deviation, frequency and percentage were computed to describe the characteristics of participants. Bivariate and multivariable analysis were done in the binary logistic regression model to identify factors. The assumptions of the binary logistic regression model were checked. A P-value<0.25 in the bi-variate analysis was considered to be a candidate variable for the final model. The Hosmer-Lemeshow goodness fitness was done to check model fitness.

Multicollinearity among independent variables was checked by the variance inflation factor (VIF). The adjusted odds ratio (AOR) with a 95% confidence interval (CI) was computed to determine the level of significance. A statistical significance was declared at a P-value< 0.05. The result was presented by using tables and figures.

### Ethical considerations

Ethical approval was granted by the Institutional Research Ethics Review Board (IRB/1222/2022) of Arba Minch University College of Medicine and Health Science. All the study participants were assured about the anonymity of the data, informed about the purpose of the study, the variety of information needed from them and informed that they were free to refuse or accept the interview. Informed verbal consent was obtained from all study participants by reading the entire consent form for the study participants and the participants’ who were agreed to verbally read the information sheet and sign the consent form to gave their permission. Beside this, the data collectors continued with the interview process for study participants’ who were agreed to participate in the study.

## 3. Results

### Socio-demographic and economic characteristics

The current study included four hundred seven postpartum mothers who were voluntarily involved. Of the study participants, 255 (62.6%) were within the age group of 21–34 years; the mean (±SD) age of the respondents was 27.34 (±6) years. One hundred forty-four (35.4%) had no formal education; 281 (69%) were housewives, and 397 (97.5%) were married. In this study, one hundred ninety (46.7%) mothers were reside rural (Table 1).

**Table 1:** Socio-demographic and economic characteristics of participants in Southeast Ethiopia, 2022 (N = 407).

Variables	Frequency	Percentage (%)
**Mother’s Education**		
Cannot read and write	144	35.4
Can read and write	57	14
Grade 1–8	97	23.8
Grade 9–12	64	15.7
**Age(in completed years)**		
15–20	74	18.2
21–34	255	62.6
≥35	78	19.2
Tertiary and above	45	11.1
**Residence**		
Urban	217	53.3
Rural	190	46.7
**Mother’s Occupation**		
Merchant	48	11.8
Government Employee	41	10.1
House wife	281	69
Private	25	6.1
Other^α^	12	3
**Marital status**		
Married	397	97.5
Others^β^	10	2.5
**Husband’s Occupation**		
Private	75	18.4
Government employee	72	17.7
Farmer	169	41.5
Merchant	91	22.4
**Husband’s Education**		
Cannot read and write	101	24.8
Can read and write	71	17.4
primary (grade 1–8)	81	19.9
Secondary(grade 9–12)	80	19.7
Tertiary and Above	74	18.2
**Husband’s Occupation**		
Farmer	169	41.5
Merchant	91	22.4
Government employee	72	17.7
Private	75	18.4
**House hold average monthly Income (ETB)**		
Greater than or equal to 2000	257	63
1001–1999	36	9
Less than or equal to 1000	114	28

^α^ -Farmer, Students, ETB-Ethiopia Birr

^β-^single, divorced, widowed

### Obstetrics care related characteristics

One hundred ninety one (65.2%) postnatal mothers had three or more living children. One hundred thirty-seven (46.8%) of the respondents had a history of home birth and 242 (82.6%) had a history of spontaneous vaginal delivery. One hundred ninety-four (47.7%) mothers came to the delivery ward through a referral from other health facilities, 184 (45.2%) from home and from the antenatal care ward 29 (7.1%) to give birth in the hospitals. The current pregnancy outcome was alive births for 388 (95.3%). Moreover, 118 (29%) of mothers developed complications after delivery and bleeding accounted for 15.7% of them (Table 2).

**Table 2:** Obstetric characteristics of participants in Southeast Ethiopia, 2022 (n = 407).

Variable		Frequency	Percentage (%)
**Gravidity**			
Grand multigravida	127		31.2
Multigravida	166		40.8
Primigravida	114		28.0
**Readiness to deliver in health facility**			
Yes	318		78.1
No	89		21.9
**Labor start**			
Day	160		39.3
Night	247		60.7
**Current mode of delivery**			
Spontaneous Vaginal delivery	241		59.2
Instrumental delivery	59		14.5
Cesarean Section	107		26.3
**Birth weight of Baby**			
Less than 2500g	35		8.6
2500-4000g	341		83.8
Greater than or equal to 4000g	31		7.6
**Type of complication**			
Severe vaginal bleeding	64		15.7
High blood pressure	47		11.5
Severe headache	35		8.6
Fever	18		4.4

### Prevalence of maternal delay in reaching health facilities

In this study, the prevalence of delay in reaching health facilities was 38.1% (95% CI = 33.3, 43).

### Factors associated with delay in reaching health facilities

Of a total of seven variables entered into multivariable analysis, four variables were statistically significant with a second delay at p< 0.05. The second delay was independently associated with monthly income, distance, referred from other health facility, and delay one.

The delay in reaching the healthcare facility was 1.87 times higher among mothers whose monthly income was below or equal to 1000.00 ETB (AOR = 1.87;95% CI:1.12 to 3.14) than among those whose monthly income was above 1000.00 ETB. Mothers who lived more than five kilometers from a healthcare facility were four times likely to delay in reaching a healthcare facility (AOR = 4.35;95% CI: 2.65,7.14) than those who lived within five kilometers.

The women who had been referred from other health facilities were 1.8 times more likely to delay in reaching the health facility during emergency obstetric care (AOR = 1.77; 95% CI: 1.01, 3.11) than those who had not been referred. The odds of mothers who had experienced delay one in reaching health care facility during emergency obstetric care services were 2.9 (AOR = 2.9; 95%CI:1.70, 4.93) as compared with those who had not experienced delay one (Table 3).

**Table 3:** Factors associated with delay in reaching health facilities among mothers who gave birth in Bale and east Bale zones, Oromia region, Southeast Ethiopia, 2022 (n = 407).

Variables	second maternal delay		COR[95%CI]	AOR[95%CI]	P value
	**Yes**	**No**			
**Residence**					
Rural	79(41.6%)	111(58.4)	1.32(0.88,1.97)	1.14(0.67,1.97)	0.622
Urban	76(35%)	141(65%)	1	1	
**House hold average monthly income(ETB)**					
≤1000	58(50.9%)	56(49.1%)	2.21(1.41,3.47)	1.87(1.12,3.14)*	0.018
1001–1999	15(41.7%)	21(58.3%)	1.52(0.75,3.11)	1.55(0.69,3.48)	0.282
≥2000	82(31.9%)	175(68.1)	1	1	
**Public transport available to go health facility**					
No	48(46.2%)	56(53.8%)	1.57(0.99,2.47)	1.51(0.86,2.66)	0.152
Yes	107(35.3%)	196(64.7)	1	1	
**Means of transportation**					
Ambulance	49(45%)	60(55%)	1	1	
Walking	21(31.3%)	46(68.7%)	0.56(0.29,1.06)	0.73(0.31,1.69)	0.456
Public T	85(36.8%)	146(63.2)	0.71(0.45,1.13)	0.82(0.46,1.48)	0.514
**Distance to reaching to nearby health facility**					
>5km	100(61.7%)	62(38.3%)	5.57(3.60,8.62)	4.35(2.65,7.14)*	<0.001
≤5km	55(22.4%)	190(77.6)	1	1	
**Referred from other health facility**					
Yes	87(44.4%)	109(55.6)	1.68(1.12,2.51)	1.77(1.01,3.11)*	0.048
No	68(32.2%)	143(67.8)	1	1	
**Delay one**					
Yes	72(60.5%)	47(39.5%)	3.78(2.42,5.92)	2.90(1.70,4.93*	<0.001
No	83(28.8%)	205(71.2)	1	1	

(* = a statistically significant variable at p< 0.05). ETB-Ethiopia Birr, T-transport)

## 4. Discussions

Maternal delays were an incomprehensible problem that affects women and can result in possibly fatal situations. The main cause of maternal mortality was the presence of these delays, along with obstetric complications. There was, however, limited data on the delay in reaching health facilities in the study area. Therefore, the aim of this study was to assess the magnitude of delay in reaching health facilities for emergency obstetric care and associated factors among postnatal mothers in the Bale and east Bale zones.

The finding of this study revealed that the magnitude of maternal delay in reaching health facilities during utilization of emergency obstetric care service was 38.1 percent (95% CI: 33.3, 43). This study was in line with a study conducted in Oromia region 37.7% [8]. This implies that there was delay in reaching health facilities at community level. However, this result was higher than the findings from studies in the Hadiya zone 29.7% [3] and Arsi zone 30.1% [12]. The difference could be due to poor road conditions, inadequate ambulance service and low economic status of the women. On the other hand, the findings of this study were lower than the studies done in India 54.5% [11], Nepal 52% [17], Mozambique 40.4% [18] Egypt 50.2% [10] and Southern Ethiopia 43.2% [16],44% [13] and 48% [19]. This discrepancies was might be time of the study, place, populations, sample size of the study and way of person’s life.

This study found that women who travel more than five kilometers from health facilities were more likely to delay in reaching health care facilities compared to those living within five kilometers. This result was supported by the studies conducted in southern Ethiopia [3, 13]. The possible reasons might be a place of residence, road and/or transport inaccessibility and the absence of a functioning nearby health facility for emergency obstetric care services. As the health facility was too far, the laboring mothers might be delayed in getting to the medical institution, miss vital emergency obstetric care treatments and develop a life-threatening complication, which could cause maternal morbidity and mortality. This implies that women have difficulty to obtain transportation services [13].

According to this study mothers who were referred from other health care facilities were more likely to be delayed during emergency obstetric care in reaching the hospital than non-referred mothers (a woman who was not referred by another health care facility and gave birth at the hospital). This result was similar with a study conducted in a northern Ethiopia [20]. The possible reasons were that they had the same study design and sociodemographic characteristics as the study participants.

An average monthly income was significantly associated with the second delay in reaching a healthcare facility. This finding was similar with a study undertaken in the Oromia region [12]. This could be explained by the women’s lack of participation in income-generating activities, which may have directly caused the problem of not having access to transportation services even if transportation services were available, as well as needing more relatives to accompany them at times of referral in case of emergencies.

According to this finding, delay one was significantly associated with delay in reaching health facilities. This was supported by the study done in the Southern Nation, Nationalities and Peoples region (SNNP) region [13]. This might be a result of participants’ shared socio-demographic characteristics and study design. This implies that there was delay at family unit, where decision made to go health facilities to seek institutional deliveries.

### Strengths of the study

Data was collected using the ODK collect application which increase the completeness, accuracy as well as quality of data. For this delay, each variable was managed independently to control the effect of confounders that prevented bias from being introduced at the analysis stage.

### Limitations of the study

Since this delay was measured by time based on client responses’ estimations, it can be overestimated or underestimated. Besides, since an interviewer-administered questionnaire was used to collect data that may be subject to recall bias. Furthermore, this study employed a cross-sectional design that failed to differentiate between cause and effect relationships.

## 5. Conclusions

This study showed that the magnitude of maternal delay in reaching health facilities for utilization of emergency obstetric care services in the study area was high. Distance, monthly income, a referral from another health facility and first delay were predictors of second maternal delay. The government should be promoting road availability, quality, closeness, and transportation mechanisms to health facilities and the community. The health sector would promote income-generating mechanisms for mothers, and hospitals would be strengthening collaboration with primary health care sub-facilities to improve a maternal referral system such as a liaison office.
